# In TCR-Stimulated T-cells, N-ras Regulates Specific Genes and Signal Transduction Pathways

**DOI:** 10.1371/journal.pone.0063193

**Published:** 2013-06-03

**Authors:** Stephen J. Lynch, Jiri Zavadil, Angel Pellicer

**Affiliations:** 1 Department of Pathology, New York University Langone School of Medicine, New York, New York, United States of America; 2 Department of Pathology, N.Y.U. Cancer Institute and Center for Health Informatics and Bioinformatics, New York University Langone Medical Center, New York, New York, United States of America; 3 New York University Cancer Institute, New York University Langone School of Medicine, New York, New York, United States of America; University of Rome, Italy

## Abstract

It has been recently shown that N-ras plays a preferential role in immune cell development and function; specifically: N-ras, but not H-ras or K-ras, could be activated at and signal from the Golgi membrane of immune cells following a low level T-cell receptor stimulus. The goal of our studies was to test the hypothesis that N-ras and H-ras played distinct roles in immune cells at the level of the transcriptome. First, we showed via mRNA expression profiling that there were over four hundred genes that were uniquely differentially regulated either by N-ras or H-ras, which provided strong evidence in favor of the hypothesis that N-ras and H-ras have distinct functions in immune cells. We next characterized the genes that were differentially regulated by N-ras in T cells following a low-level T-cell receptor stimulus. Of the large pool of candidate genes that were differentially regulated by N-ras downstream of TCR ligation, four genes were verified in qRT-PCR-based validation experiments (*Dntt*, *Slc9a6*, *Chst1*, and *Lars2*). Finally, although there was little overlap between individual genes that were regulated by N-ras in unstimulated thymocytes and stimulated CD4^+^ T-cells, there was a nearly complete correspondence between the signaling pathways that were regulated by N-ras in these two immune cell types.

## Introduction

The three primary Ras isoforms (N-ras, H-ras and K-ras) share a high degree of structural and functional similarity, which initially led some researchers to propose that the Ras proteins are functionally redundant. However, it has been shown that the Ras isoforms are functionally distinct: (1) there are differences in both the levels and temporal dynamics of expression of the *Ras* mRNAs [1], (2) the Ras isoform phenotypes of knockout mice (KO) differ [2]–[5], (3) point mutations that lead to constitutive activation of each of the *Ras* proto-oncogenes lead to the development of tumors in different tissue types [6], and (4) the Ras isoforms were found to have different affinities for both upstream modulators and downstream effectors [7]–[9].

Each of the Ras isoforms has also been shown to associate with cellular membranes through different mechanisms. The membrane association of N-ras and H-ras has been shown to be dependent upon the addition of palmitate moieties to C-terminal cysteines, whereas K-ras does not get palmitoylated, and instead associates with cellular membranes through a C-terminal polybasic lysine motif (Lys_175_-Lys_180_). Although N-ras and H-ras are both palmitoylated at their C-termini, N-ras is singly palmitoylated at C181, whereas H-ras is doubly palmitoylated at C181 and C184.

Ras-mediated signal transduction in T-cells is initiated through ligation of the T-cell receptor (TCR) by antigen:MHC complexes present on the surface of the antigen presenting cell (APC) at a specialized cellular structure known as the immunological synapse [10]. Full TCR-mediated signaling leads to recruitment of Grb2 and Sos, that have been shown to be directly responsible for the activation of Ras at the plasma membrane (PM) in T-cells. A number of signal transduction pathways are downstream of activated Ras in T-cells [11]–[13].

N-ras has been postulated as the Ras isoform that plays the most prominent role in the development of the hematopoietic system [14]. Early studies indicated that *N-ras* mRNA was preferentially expressed in the thymus, implying that N-ras might play a functional role in the development of immune cells in this organ [1]. In addition, constitutive activation of N-ras, both in patient populations and in transgenic mice models, was linked to the development of a variety of hematopoietic malignancies, including: acute myeloid leukemia, lymphoblastic T-cell lymphomas, cleaved B-cell lymphomas, and myelodysplastic syndrome [15]–[17]. In contrast, transgenic mice with constitutive activation of H-ras did not develop those malignancies [18]. Finally, a reexamination of the N-ras KO mouse revealed that there were significant decreases in the size of the CD8^+^ thymocyte single positive population, as well as decreases in the numbers of cells associated with T-cell positive selection, implying that T-cell positive selection was defective in the N-ras KO [14]. In addition, the N-ras KO mouse was highly susceptible to an influenza challenge, with N-ras KO mice succumbing much earlier to lower doses of influenza than their wild type (WT) counterparts [14].

Previous studies by Philip's group and others had provided evidence that N-ras and H-ras localized to the Golgi membrane, where they were able to function as nuclei for downstream signaling [19]–[21]. This initial work was extended in a later collaborative study between the Philips and Pellicer groups, where it was shown that in Jurkat T-cells a low-level TCR stimulation (1 μg/ml α-CD3e and α-CD28 antibodies) induced the activation of endogenous N-ras, but not endogenous H-ras or K-ras [22]. Furthermore, N-ras activation occured at the Golgi membrane with this TCR stimulus paradigm [22]. It was also shown that the different palmitoylation states of N-ras and H-ras were important determinants of the specificity of Ras signaling from the Golgi membrane following a low-level TCR stimulus. In these experiments, a mono-palmitoylated H-ras mutant (H-rasC184L, a.k.a. H-ras-Palm*N*) was activated at and signaled from the Golgi membrane following a low-level TCR stimulus, whereas a doubly-palmitoylated N-ras mutant (N-rasL184C, a.k.a. N-ras-Palm*H*) was not activated at the Golgi.

The goal of our studies was to examine the functional specificity of the N- and H-ras isoforms at the level of the transcriptome. To this end, we first utilized mRNA expression profiling to characterize N-ras- and H-ras-specific gene regulation in unstimulated thymocytes. In a second set of experiments, we utilized RNA differential display to determine the N-ras-specific transcriptome in TCR-stimulated T-cells. We have identified genes and pathways specifically activated by N-ras in both settings.

## Materials and Methods

### Mice

The generation of both the N-ras KO mouse and the H-ras null mouse have been described in detail elsewhere [2], [5]. The genetic background of both KO mouse strains was 129/SJ. All WT mice used in our experiments were littermates of N-ras or H-ras KO mice.

### Plasmids

The expression constructs that were used were derived from a MIGR1 retroviral vector [23], which was a gift from Iannis Aifantis' laboratory (Department of Pathology – N.Y.U. Langone Medical Center). Human N-ras, H-ras, or N-ras-Palm*H* sequences were inserted between the BamHI and HindIII sites of the Multiple Cloning Site (MCS) of MIGR1 via a PCR-based cloning strategy. Since MIGR1 lacks the *gag*, *pol*, and *env* genes required for retroviral replication and packaging, transduction of murine cell lines with MIGR1 required the cotransfection of a psi ecotropic packaging vector.

### RNA isolation

Total RNA was isolated from cells using Trizol reagent (Invitrogen), following a protocol obtained from the manufacturer. For the first set of microarray experiments, total RNA was isolated from thymocytes from 6 week-old N-ras KO, H-ras KO and WT mice. For the second set of microarray experiments, splenocytes were isolated from 6–20 week old WT and N-ras KO mice, and following incubation of the splenocytes with ACK buffer (to lyse red blood cells), CD4^+^ T cells were isolated by FACS using an APC-conjugated rat anti-mouse CD4 antibody (ebioscience – clone RM4-5). Following FACS, the CD4^+^ T cells were spun down and resuspended in T-cell growth media, and the sorted cells were split into two groups: one that received an overnight low-level TCR stimulus (1 μg/ml α-CD3e and αCD28), and a second group that was left unstimulated overnight. RNA was then isolated from the stimulated and unstimulated CD4^+^ T cells using Trizol reagent.

In both microarray experiments, the RNA pellet was briefly air-dried, and was resuspended in 20 μl of nuclease-free water (Ambion). The concentration, purity, and integrity of total RNA were determined by NanoDrop ND-1000 and Agilent 2100 Bioanalyzer.

### cDNA synthesis

cDNA was synthesized from 5–8 μg of total RNA using the SuperScript Double-Stranded cDNA Synthesis Kit from Life Technologies, according to the manufacturer's instructions. A T7-(dT) 24 primer (5′-GGC CAG TGA ATT GTA ATA CGA CTC ACT ATA GGG AGG CGG-(dT)_24_ – HPLC purified) was mixed with the RNA during the first stage of the cDNA synthesis. The resulting cDNA was purified via phenol:chloroform:isoamyl alcohol extraction.

### RNA Microarray Hybridization

cRNA was synthesized from cDNA using the Bioarray HighYield RNA Transcript Labeling Kit (T7) (Enzo Life Sciences). The product of the cRNA synthesis reaction was purified using a RNeasy Mini Kit (Qiagen) according to the manufacturer's instructions. RNA quality and quantity was assessed using an Agilent 2100 Bioanalyzer and a Nanodrop ND-100 (Agilent Technologies). If the cRNA yield was low, cRNA was amplified using a WT-Ovation Pico RNA Amplification kit (Nugen) prior to microarray hybridization. Fragmentation and labeling were performed using the FL Ovation cDNA Biotin Module V2 (Nugen) according to the manufacturer's instructions. The samples were subsequently hybridized on Mouse Genome 430A 2.0 arrays (Affymetrix) – these arrays contained target probes for approximately 14,000 well-characterized mouse genes.

### Microarray Data Analysis

The Genespring GX11 software was used for analysis of the raw microarray data. The expression value of each probe set was determined after quantile normalization using the robust microarray analysis (RMA)-16 algorithm and baseline transformation to the median levels of control samples. An unpaired Student's *t* test was used to determine the statistical significance of differences observed, where values of *p*<0.05 were considered significant. Unless otherwise stated, genes with a fold-change (F.C.) of at least 1.5 were considered to be specifically upregulated or downregulated in the microarray analysis. Gene ontology of target genes was conducted with the Functional Annotation Tool of the NIH DAVID database (http://www.ncbi.nlm.nih.gov/pubmed/19131956?dopt= Abstract).

### qRT-PCR-based Validation of Candidate Genes

Candidate genes from the second set of RNA differential display experiments were further tested via two sequential rounds of qRT-PCR-based validations. In the first round of validation experiments, qRT-PCR was performed using cDNA derived from WT CD4^+^ T-cells or N-ras KO CD4^+^ T-cells transduced with empty vector. The second round of validation experiments included qRT-PCR reactions performed with cDNA synthesized from N-ras KO CD4^+^ T-cells transduced with either MIGR1-N-ras, MIGR1-H-ras or MIGR1-N-ras-Palm*H*. The protocol used for the retroviral transduction of CD4^+^ T cells is detailed in the Supporting Information (Protocol S1). cDNA was synthesized from total RNA isolated from retrovirally transduced CD4^+^ T cells using a iScript cDNA Synthesis Kit (Biorad), according to the manufacturer's instructions. The resultant cDNA was diluted 1∶3 with nuclease-free water prior to qRT-PCR. All qRT-PCR reactions used the iQ SYBR Green Supermix (Biorad) and were performed in a Biorad iCycler, which was coupled to a Biorad MyiQ Single Color Real-Time PCR Detection System. The qRT-PCR primers used for testing candidate genes from the microarray are listed in Table S1. *Lrrfip1* was shown to have a fold change of approximately 1 across all potential pair-wise microarray data comparisons, and this gene was therefore used as a normalization control for the qRT-PCR analysis. The relevant data were normalized to the [KO + MIGR1] condition.

### Unstimulated WT, H-ras KO and N-ras KO Thymocyte Microarray

In this experiment, mRNA was isolated from thymocytes from either WT, N-ras KO or H-ras KO mice (see above for protocol), and cDNA synthesis and RNA microarray hybridization was performed as described above. cRNA was hybridized to separate Affymetrix gene chips for each of the three experimental conditions. The microarray hybridizations were not repeated with cRNA derived from additional mice for these experiments. As our goal in these experiments was merely to test the hypothesis that N-ras and H-ras regulate different sets of genes in immune cells, we did not attempt to validate any candidate genes from this microarray.

### TCR-stimulated N-ras KO CD4^+^ T-cell Microarray

As described above, mRNA was isolated from CD4^+^ T-cells derived from WT or N-ras KO mice that had either been treated with a low-level TCR stimulus (1 μg/ml α-CD3e and α-CD28) overnight or had been left untreated. cDNA synthesis and RNA microarray hybridization was performed as described above. One mouse was used for each of the four experimental conditions: 1. WT without stimulation, 2. WT with stimulation, 3. N-ras KO without stimulation, and 4. N-ras KO with stimulation), and cRNA was hybridized to separate Affymetrix gene chips for each of the experimental conditions. The entire experiment, from RNA analysis through cDNA synthesis and microarray hybridization, was repeated three times. For each of the repetitions of the microarray experiment, RNA was isolated from a different mouse each time for each experimental condition. Candidate genes from this microarray experiment were tested as described above.

### Submission of Microarray Data

The data discussed in this publication have been deposited in NCBI's Gene Expression Omnibus and are accessible through GEO Series accession number GSE45739 (http://www.ncbi.nlm.nih.gov/geo/query/acc.cgi?acc=GSE45739).

## Results

### N-ras and H-ras Regulate the Expression of Different Genes in Immune Cells

Although our ultimate goal was to define the N-ras specific transcriptome in T-cells in the context of a low-level TCR stimulus, it was first necessary that we define the N-ras- and H-ras-specific transcriptomes in unstimulated thymocytes. Thymocytes were isolated from 6 week old N-ras KO, H-ras KO and WT mice, and differential display expression analyses were performed with cDNA derived from total RNA isolated from these mice. The microarray data sets were compared between the WT condition and either the N-ras KO or the H-ras KO conditions. In the [WT] *vs.* [N-ras KO] comparison, 303 genes were differentially regulated by N-ras (F.C. ≥1.5, *p*-value <0.05); of these genes, 167 were upregulated, and 136 genes were downregulated (Table S3). In the [WT] *vs.* [H-ras KO] comparison, 367 genes were differentially regulated by H-ras (F.C. ≥1.5, *p*-value <0.05), with 212 genes upregulated and 155 genes downregulated in this comparison (Table S4). To determine if N-ras and H-ras regulate different sets of genes in thymocytes, a comparison was made between the set of genes that were differentially regulated by N-ras in the [WT] *vs.* [N-ras KO] comparison and the set of genes that were differentially regulated by H-ras in the [WT] *vs.* [H-ras KO] comparison.

**Figure 1:**
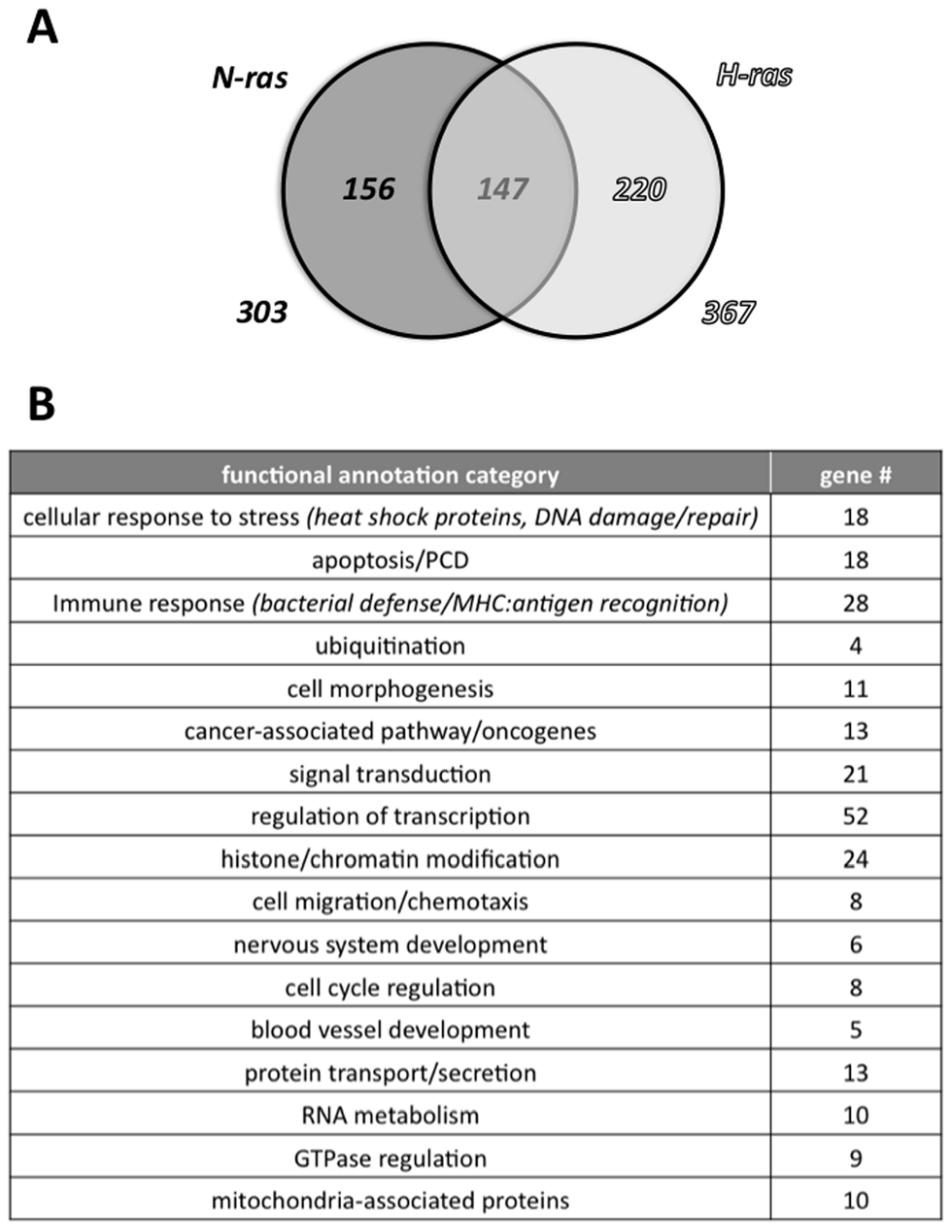
Genes modulated by N-ras and/or H-ras in unstimulated thymocytes. (A) Venn diagram of the numbers of genes that were differentially regulated in the [WT] *vs.* [N-ras KO] and the [WT] *vs*. [H-ras KO] microarray data comparisons. In addition, a description of the numbers of genes that were similarly regulated by H-ras and by N-ras is shown, and the numbers of genes that were uniquely differentially regulated by either N-ras or H-ras is also given. (B) Results of a medium stringency DAVID database analysis of the genes differentially regulated by N-ras in the unstimulated thymocyte microarray experiments. For each of the functional annotation categories, the numbers of differentially regulated genes in that category are also shown. Note that the functional categories shown are, in some cases, derived from a combination of multiple functional annotation categories from the original DAVID database analysis. RNA from one mouse was used for each of the three experimental conditions.

Of the total of 523 genes that were differentially regulated by N-ras and/or by H-ras, 147 of these exhibited differential regulation by both Ras isoforms (see Figure 1A). Note that the candidate genes that are in bold-faced font in Tables S3 and S4 are genes that were differentially regulated by both Ras isoforms in this array. These 147 genes were presumably part of pathways that are fundamental components of general Ras signaling and function, and included in these were genes for the transcription factors Creb-1, Fos, and Jun; as well as genes for Gnb1 (guanine nucleotide signaling) and Gadd45-α (growth arrest in response to DNA damage). These 147 genes were skewed somewhat towards upregulated genes (61% upregulated, 39% downregulated).

As Figure 1A illustrates, of the 303 genes that were differentially regulated by N-ras, 156 genes were ‘uniquely differentially regulated’, in that they were not also differentially regulated by H-ras. In a similar fashion, for the 367 genes differentially regulated by H-ras, 220 of these genes were uniquely differentially regulated by H-ras. The genes from Tables S3 and S4 that are not in bold-faced font are those genes that were uniquely differentially regulated by the respective Ras isoform in this microarray experiment. It should be noted that we did not observe any compensatory upregulation of H-ras expression in N-ras KO cells, and we also did not see an upregulation of N-ras in H-ras KO cells. Genes that were differentially regulated by one Ras isoform but not the other presumably represent factors that play fundamental roles in Ras isoform-specific signaling and function. Overall, the fact that there were nearly four hundred genes that were uniquely differentially regulated by N-ras or by H-ras clearly supports the hypothesis that the two Ras isoforms regulate different sets of genes in immune cells.

The NIH DAVID database was used to group the 303 genes that were differentially regulated by N-ras into a total of 17 major functional annotation categories (see Figure 1B). It should be noted that this DAVID database analysis utilized the 303 genes that were differentially regulated by N-ras, rather than using the smaller pool of 156 genes that had been shown to be uniquely differentially regulated by N-ras (i.e. were not also differentially regulated by H-ras). This was done to facilitate a comparison to the DAVID database analysis results for the second microarray, where we did not exclude genes that were also differentially regulated by H-ras (see below). N-ras had been previously shown to play roles in immune response, signal transduction, cancer-associated pathways/oncogenes, and cell proliferation, and these functional annotation groups were therefore expected. Other functional annotation categories, such as: histone/chromatin modification, ubiquitination or RNA metabolism were not expected, however, these novel pathways downstream of N-ras clearly warrant further investigation.

### N-ras-Specific Transcriptome in CD4^+^ T-cells Following a Low-level TCR Stimulus

**Figure 2:**
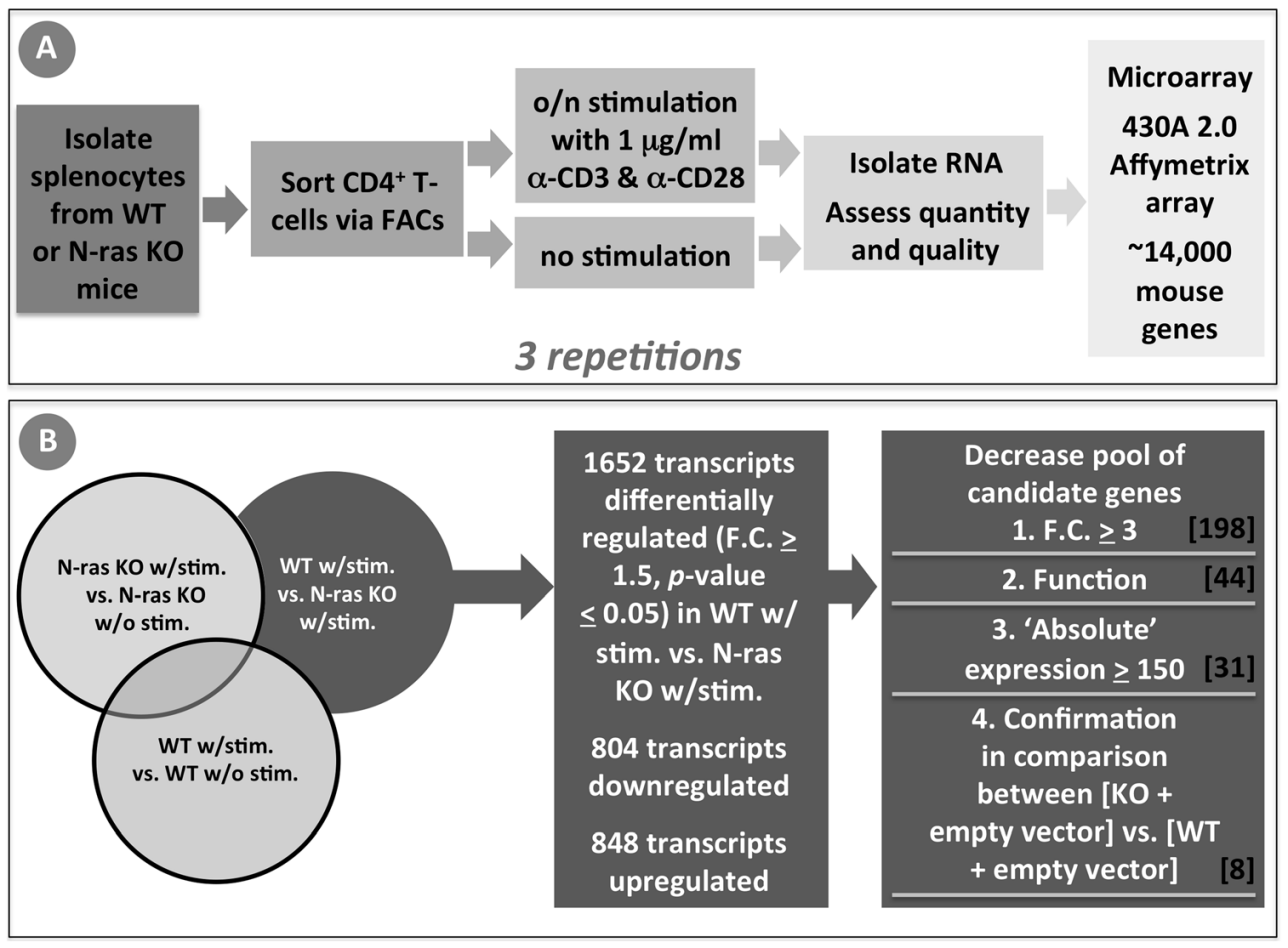
Flowchart of the RNA differential display experiments to determine the N-ras-specific transcriptome in TCR-stimulated CD4^+^ T-cells. (A) Flowchart of the second set of RNA differential display experiments. (B) Schematic illustrating the process used to further define differentially regulated genes from the microarray as potential candidate genes for subsequent testing.

In order to determine the N-ras-specific transcriptome in TCR stimulated T-cells, a RNA differential display analysis was performed with RNA derived from the following four experimental conditions: (1) WT CD4^+^ T-cells incubated overnight without a TCR stimulus, (2) WT CD4^+^ T-cells incubated overnight with a low-level TCR stimulus (1 μg/ml α-CD3e and α-CD28), (3) N-ras KO CD4^+^ T-cells incubated overnight without a TCR stimulus, and (4) N-ras KO CD4^+^ T-cells incubated overnight with a low-level TCR stimulus. Since we were interested primarily in genes that were differentially regulated by N-ras following a low-level TCR stimulus, our microarray data comparison was between data from TCR-stimulated, WT CD4^+^ T-cells and from TCR-stimulated, N-ras KO CD4^+^ T-cells. Genes that were differentially regulated in the comparison between stimulated N-ras KO CD4^+^ T-cells and unstimulated N-ras KO CD4^+^ T-cells, as well as those genes that were differentially regulated in the comparison between stimulated WT CD4^+^ T-cells and unstimulated WT CD4^+^ T-cells were excluded from this analysis. Figure 2A contains a flowchart of the second set of microarray experiments and Figure 2B describes the process through which we incrementally defined potential candidate genes from the array. The entire experiment was repeated three times, with all three repetitions being used to compile the list of differentially regulated transcripts.

**Figure 3:**
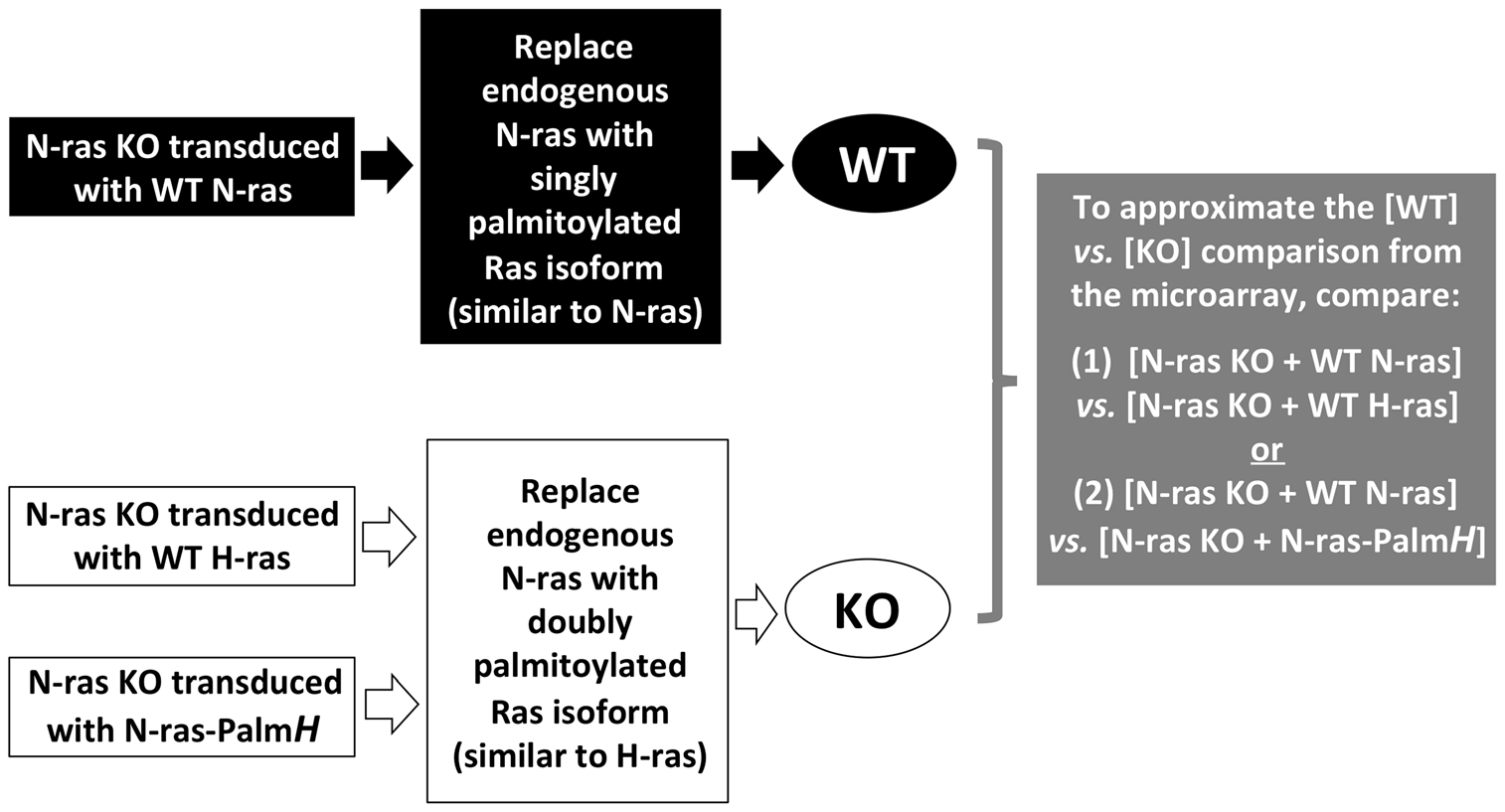
Schematic of the rationale and the experimental design of qRT-PCR-based validation experiments that were performed with candidate genes from the second set of microarray experiments. The assumption behind these experiments was that over-expression of WT N-ras in N-ras KO CD4^+^ T-cells should be able to reconstitute or rescue the gene expression of the candidate genes as seen in WT cells. In contrast, over-expression of WT H-ras in N-ras KO CD4^+^ T-cells should not be able to rescue the expression of these candidate genes, and N-ras KO cells over-expressing WT H-ras should therefore be most similar to N-ras KO cells in their pattern of gene expression. From a palmitoylation state perspective, N-ras-Palm*H* was similar to WT H-ras, and one would therefore not expect that over-expression of N-ras-Palm*H* in the N-ras KO background would be able to rescue the expression of candidate genes downstream on N-ras.

All microarray data comparisons were consistently made between [WT] *vs.* [N-ras KO], or in the case of the subsequent rescue/validation experiments, between conditions that approximated WT and N-ras KO, such as [KO + N-ras WT] *vs.* [KO + H-ras WT] (see Figure 3). We chose to represent the microarray data comparisons in this format to simplify the interpretation of the microarray data. Genes that were found to be upregulated in this comparison were upregulated by N-ras and conversely, genes that were downregulated in this array data comparison were downregulated by N-ras.

A total of 1848 transcripts were differentially regulated in the [WT + stimulation] *vs.* [N-ras KO + stimulation] microarray data comparison (F.C. ≥1.5, *p*-value ≤0.05), and elimination of the 196 transcripts that were also differentially regulated in the [WT] *vs.* [WT + stimulation] and the [N-ras KO] *vs.* [N-ras KO + stimulation] yielded 1652 differentially regulated transcripts (see Table S5). For the differentially regulated transcripts, 819 represented clearly defined genes, whereas the other 833 represented either duplicate transcripts of the same gene, expressed sequence tags or cDNA clones. Of the 1652 transcripts, 848 were upregulated, and 804 were downregulated. A medium stringency DAVID database analysis was performed to assign the differentially regulated genes into functional annotation categories: the differentially regulated genes were grouped into a total of 18 major functional annotation categories (Table 1). As was the case for the DAVID database analysis of the data from the [WT] *vs.* [N-ras KO] unstimulated thymocyte microarray, the functional annotation categories for the second set of array experiments were both expected (cell death, immune response, cell commitment/differentiation, and signal transduction) and unexpected (RNA localization/metabolism/binding, DNA damage/repair, and chromatin binding/remodeling).

**Table 1:** Results of a medium stringency DAVID database analysis of the genes differentially regulated by N-ras in the stimulated CD4^+^ T-cell microarray experiments.

functional annotation category	total	U	D	unique	gene names of unique members of each category
cell death	16	6	10	3	Xiap, Cflar///LOC100040853, Bcl2l13
ubiquitin ligase activity	4	1	3	1	Siah2
RNA localization/metabolism/binding	6	2	4	4	Srrm1, Refbp2///Thoc4, Gemin8, Bruno14
cytoskeleton	10	2	8	6	Dctn4, Synpo, Vapa, Spag16, Cttnbp, Mtap1s
cell shape changes/morphogenesis	10	5	5	1	Prlr
blood vessels	14	6	8	5	Hey1, Wars2, P2rx4, Taz, Agtrap
cell cycle	8	4	4	1	Cdkn3
DNA damage/repair	10	4	6	2	Rad23a, Obfc2a
immune response	12	7	5	1	Cxcl13
cell commitment/differentiation	17	8	9	0	-
signal transduction (general)	44	21	23	13	Pik3r3, Ttll10, Ikbkb, Inpp5k, Prmt3, Prkacb, Asph, Il1rap, Igfbp5, B4giant2, Mgat5, Ccnb1, Impact
Ras-mediated signal transduction	5	3	2	3	Mcf2l, Xpo7, Arfgef2
transcriptional control	28	14	14	12	Zfhx3, Scmh1, Churc1, Lcor, Mef2a, Carf, Per3, Runx1t1, Nfkble, Pax9, Dnaib6, LOC100
chromatin binding/remodeling	13	8	5	3	Lin28, Ncapd2, Hist1h1e
vesicular transport	14	4	10	0	-
cell contacts	9	4	5	7	Dpp4, Tin1, Stk39, Dsg2, Mpdz, Slc12a6, Aqp3
molecular transport	12	6	6	3	Syne2, Nutf, Fras1
nervous system	26	12	14	9	Racgap1, Efhd1, Dfna5, Tcfap2a, Pcdh15, Nrxn1, Lhx8, Ret

For each of the 18 functional annotation categories listed, the number of genes that were downregulated (D), the number of genes that were upregulated (U), and the number of unique genes (i.e. genes that were not assigned to another functional annotation category) are shown. For each functional annotation category, the names of unique members of each category, as well as the candidate genes from that category that were tested in qRT-PCR-based validation experiments is also shown. Note that the functional categories shown are, in some cases, derived from a combination of multiple functional annotation categories from the original DAVID database analysis.

Since 1652 transcripts were far too many candidates to attempt to test further, we reduced our candidate gene pool by increasing the stringency of our criteria of what we considered as a differentially regulated gene (Figure 2B). Considering only clearly defined genes as potential targets left us with 819 candidates. Increasing our fold change cut-off from ≥1.5 to ≥3 left us with 198 candidate genes. In addition, we revisited the DAVID database analysis data, and eliminated genes as candidates if they did not belong to functional annotation categories consistent with previous functions ascribed to N-ras in immune cells, such as: signal trnsduction, oncogenesis, or immune response. This third reduction left us with 44 candidate genes. In addition, we ruled out any candidate genes for further qRT-PCR-based testing if they had an absolute expression value in the raw array data of less than 150. This was done because previous attempts to validate genes with an absolute expression value in the raw microarray data of less than 150 were unsuccessful, as the change in expression of these genes was below the level of sensitivity of our qRTPCR-based assay. The 31 candidate genes that remained after this final cut-off criterion are listed in Table S2.

### Comparison and Interpretation of Data from the Unstimulated Thymocyte and Stimulated CD4^+^ T-cell Microarray Experiments

In the hopes of elucidating genes whose regulation is of fundamental importance for N-ras-specific signaling and function in immune cells, we compared genes that were differentially regulated by N-ras in the unstimulated thymocyte microarray to genes that were differentially regulated by N-ras in the stimulated CD4^+^ T-cell splenocyte microarray. In both microarray experiments, there was a roughly 1∶1 ratio of genes that were upregulated and downregulated by N-ras. Only fifteen genes that were differentially regulated by N-ras in unstimulated thymocytes were also differentially regulated by N-ras in stimulated CD4^+^ T-cells (Table 2), which was not surprising, given the different cellular contexts of the two experiments.

**Table 2:** Comparison of genes modulated in unstimulated thymocytes to those modulated in T-cell splenocytes treated with a low-level TCR stimulus.

Gene	Gene name	*Unstimulated thymocytes*		*Stim. CD4+ T-cells*
		[WT] *vs.* [N-ras KO]	[WT] *vs.* [H-ras KO]	[WT] *vs*. [N-ras KO]
Agtrap	angiotensin II, type I receptor associated protein	−−		++
Camp	cathelicidin antimicrobial peptide	+	+++	−−
Cbx5	chromobox homolog 5 (Drosophila HP1a)	+		++
Kif2c	kinesin family member 2C	+		++
Klf4	Kruppel-like factor 4 (gut)	+	++	−
Mgea5	meningioma expressed antigen 5 (hyaluronidase)	−		−−
Mll3	myeloid/lymphoid or mixed-lineage leukemia 3	−		−
Ngp	neutrophilic granule protein	++	+++	−−
Pafah1b1	platelet-activating factor acetylhydrolase, isoform 1b, beta 1 subunit	−		−
Sema4a	sema domain, immunoglobulin domain (Ig), transmembrane domain (TM) and short cytoplasmic domain, (semaphorin) 4A	−		++
Skp2	S-phase kinase-associated protein 2 (p45)	−−		++
Snai1	snail homolog 1 (Drosophila)	+		−
Srrm1	serine/arginine repetitive matrix 1 (Srrm1), transcript variant 1, mRNA	−		−−
Tpp2	tripetidyl petidase II	−	−	−
Zfp106	zinc finger protein 106	−	−	++

In addition to listing the gene name and gene symbol, the last three columns of the table indicate if the gene was found to be differentially regulated in the [WT] *vs*. [N-ras KO] and [WT] *vs*. [H-ras KO] comparisons from the unstimulated thymocyte array data and/or was differentially regulated in the [WT] *vs*. [N-ras KO] comparison from the stimulated CD4^+^ T-cell array data. In the last three columns of the table: “+” indicates a F.C. >1.5, but less than 2; “++” indicates a F.C. between 2 and 5; “+++” indicates a F.C. >5; “−” indicates a F.C. <0.667, but greater than 0.5; and “−−” indicates a F.C. between 0.5 and 0.2.

Of the fifteen genes that were differentially regulated by N-ras in both arrays, we were most interested in those genes that were also: (1) regulated in the same direction by N-ras in both cell types, and (2) uniquely differentially regulated by N-ras (i.e. not also differentially regulated by H-ras) in the unstimulated thymocyte microarray. We hoped that excluding genes that were also differentially regulated by H-ras in unstimulated thymocytes would allow us to focus only on those genes that played N-ras-specific functions in immune cells. Only five of the fifteen genes met these two criteria, namely: *Cbx5, Kif2C, Mgea5, Mll3,* and *Srrm1*. With regards to the functional products of these five genes: Cbx5 is a mammalian homolog of a Drosophila protein shown to be important in post-gastrulation embryonic development, Kif2C is a kinesin protein associated with the centromeres and spindle development during cell division, Mgea5 is a cytoplasmic O-Glc NAcase enzyme, Mll3 plays a role in H3K4 methylation in the regulation of transcription by RNA polymerase II, and Srrm1 is a co-activator of pre-mRNA splicing. Overall, the five genes that were uniquely and similarly differentially regulated by N-ras in both immune cell types belonged to pathways associated with cell division, post-translational processing, and chromatin remodeling. *Mgea5* and *Mll3* were also noteworthy because these genes have been shown to play roles in oncogenesis. The fact that these five genes were both similarly and uniquely differentially regulated by N-ras in two different immune cell types implied that they might play fundamental roles in N-ras signaling and function in lymphocytes.

Comparing the functional annotation categories from the DAVID database analyses of differentially regulated genes in the [WT] *vs.* [N-ras KO] comparisons from the unstimulated thymocyte and stimulated CD4^+^ T-cell microarray experiments (compare Figure 1B to Table 1) revealed that, with a few exceptions, almost all of the functional annotation categories listed in Table 1 were common to both immune cell types. A high degree of overlap between the functional annotation categories from the separate sets of microarray experiments was somewhat unexpected, given the low level of commonality between the individual genes that were differentially regulated by N-ras in the two experiments. The fact that many of the same pathways were regulated by N-ras in two very different cellular contexts was noteworthy, because it suggests that these pathways are of fundamental importance in N-ras signaling in immune cells.

### Validation of Candidate Genes from the RNA Differential Display Comparisons between WT and N-ras KO in TCR-stimulated CD4^+^ T-cells

To test the 31 candidate genes from the second microarray experiment further, we first determined the pattern of expression of each of the candidates in a qRT-PCR-based comparison between WT *vs.* N-ras KO CD4^+^ T-cells transduced with empty vector (MIGR1). Any candidate genes that did not exhibit a similar pattern of expression in the qRT-PCR-based [WT + MIGR1] *vs.* [KO + MIGR1] comparison and in the microarray [WT + stimulation] *vs*. [KO + stimulation] comparison were ruled out for further testing. In order to replicate the stimulus paradigm that was used in the microarray, the transduced CD4^+^ T-cells were stimulated overnight with 1 μg/ml α-CD3e and α-CD28 antibodies prior to RNA isolation. The eight candidate genes that exhibited a similar pattern of regulation in the microarray comparison and in the qRT-PCR comparison between [WT + empty vector] and [KO+ empty vector] transduced T-cells are indicated in Table 3.

**Table 3:** Properties of eight candidate genes that were tested in qRT-PCR-based validation experiments.

F.C.	*p*-value (T test)	Genename	Gene title	Functional annotation categories/ proposed function	Avg. ab s. expr.	Med. abs. expr.
4.17	0.0283	Ehd1	EH-domain containing 1	signal transduction, vesicle mediated transport	319	391
4	0.0471	Chst1	carbohydrate (keratan sulfate Gal-6) sulfotransferase 1	sulfotransferaase/transferase activity, metabolic processes, inflammatory response	885	837
4	1.10E-04	Lars2	leucyl-tRNA synthetase, mitochondrial	ATP/nucleotide binding, ligase activity, aminoacylation and translation	751	747
3.7	0.0193	Mbnl3	Muscleblind-like 3 (Mbnl3, Drosophila homolog)	ion and nucleic acid binding, RNA splicing, mRNA processing	401	382
3.45	0.0298	Slc9a6	solute carrier family 9 (sodium/hydrogen exchanger), member 6 (Slc9a6)	Antiporter activity	282	270
3.03	0.0011	Ncl	nucleolin	nuclear associated – DNA/RNA binding	575	599
3.23	0.023	Glmn	glomulin, FKBP associated protein	vasculogenesis, negative regulation of T cell proliferation, regulation of cytokine secr.	231	240
0.09	0.0326	Dntt	deoxynucleotidyltransferase, terminal	permanently silenced during transition from immature to mature thymo with TCR signaling	166	181

For each of the candidate genes, the fold change (F.C.), *p*-value, gene name, gene title, functional annotation category/proposed function, average absolute expression values in the raw microarray data, and median absolute expression values in the raw array data are listed.

The goal of the second round of qRT-PCR validation experiments was to further test the candidate genes by performing a rescue experiment. The assumption behind these experiments was that if a candidate gene was truly regulated by N-ras, then over-expression of WT N-ras in N-ras KO CD4^+^ T-cells should rescue the expression pattern of these genes, whereas over-expression of WT H-ras in N-ras KO CD4^+^ T-cells should not be able to rescue the expression pattern of these candidates (see Figure 3). The second round of validation experiments included qRT-PCR reactions that were performed with cDNA derived from either N-ras KO CD4^+^ T-cells transduced with either MIGR1-N-ras or MIGR1-H-ras. To recapitulate the [WT] *vs.* [N-ras KO] comparison made in the initial microarray analysis, qRT-PCR data comparisons between the [KO + WT N-ras] and [KO + WT H-ras] conditions were made.

**Figure 4:**
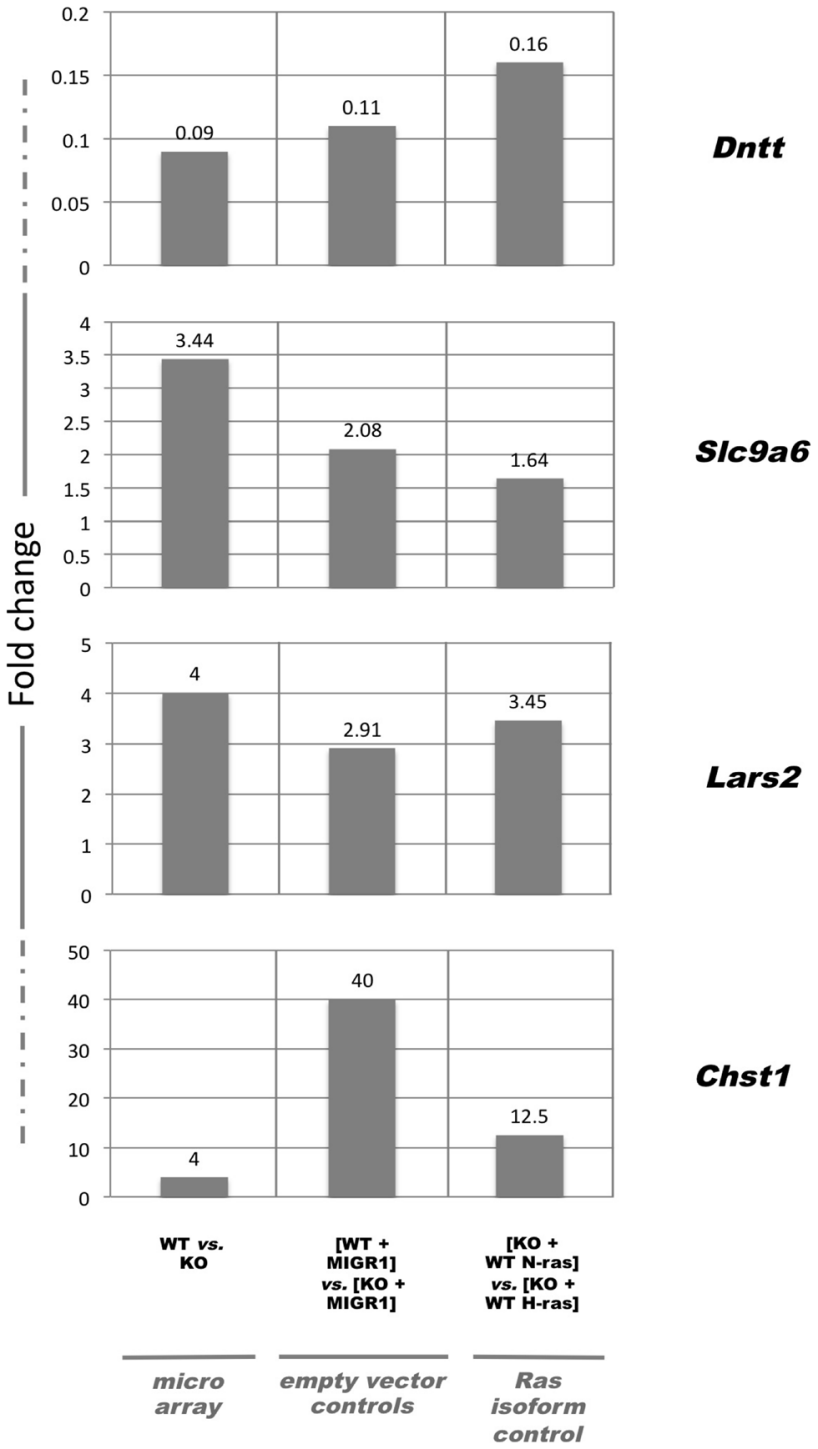
Four of the candidate genes from the second set of microarray experiments exhibited similar patterns of expression in the microarray, in the empty vector controls, and in the Ras isoform transduction validations. Graphical representation of the fold-changes for *Dntt, Slc9a6*, *Lars2*, and *Chst1* in the microarray, in the empty vector control experiments and in the Ras isoform transduction validation experiments. For each candidate gene, the fold change in the [WT] *vs.* [KO] microarray comparison, in the [WT + MIGR1] *vs.* [KO + MIGR1] empty vector control experiment, and in the [KO + WT N-ras] *vs.* [KO + WT H-ras] and [KO + WT N-ras] *vs.* [KO + N-ras-Palm*H*] Ras isoform transduction validation experiments are shown. The entire experiment, from RNA analysis through cDNA synthesis and microarray hybridization, was repeated three times, and for each repetition of the experiment, RNA was isolated from a different mouse. The relevant data were normalized to the [KO + MIGR1] condition.

Of the eight strong candidate genes that were tested in the second round of qRT-PCR-based validations, four genes had expression patterns that were similar to what had been seen in both the microarray experiments and in the empty vector control transduction experiments (see Figure 4). The other four candidate genes either exhibited patterns of expression in the N-ras transduction experiments that were not consistent with their expression patterns in the microarray and in the empty vector transduction experiment, or were found to be unchanged in the [KO + WT N-ras] *vs*. [KO + WT H-ras] q-RTPCR-based comparison. The four validated candidates were: *Slc9a6*, which encodes for NHE6, a transmembrane domain Na^+^/H^+^ ion exchanger; *Dntt*, which encodes for the Tdt enzyme, which adds nontemplate nucleotides to splice junctions during V(D)J recombination of B-cell and T-cell receptors; *Chst1* which encodes for the enzyme CHST1 (carbohydrate sulfotransferase 1), a Golgi-localized, transmembrane domain sulfotransferase that modifies lipids and proteins through sulfonation; and *Lars2*, which encodes for the LARS2 (human leucyl-tRNA synthetase 2, mitochondrial) enzyme, which had been shown to catalyze the charging of mitochondrial tRNA^Leu(UUR)^ with leucine during mitochondrial-specific protein synthesis. The potential functional relevance of these genes is explored at greater length in the Discussion.

In order to test the hypothesis that the palmitoylation state of the Ras isoform determines their transcriptional specificity, the second group of validation experiments included qRT-PCR reactions performed with cDNA derived from N-ras KO CD4^+^ T-cells transduced with the MIGR1-N-ras-Palm*H* over-expression construct. From a palmitoylation state perspective, N-ras-Palm*H* is similar to WT H-ras, and the [KO + WT N-ras] *vs.* [KO + N-ras-Palm*H*] qRT-PCR data comparison was therefore similar to the [WT] *vs.* [KO] comparison that was made in the microarray (see Figure 3). The four validated candidate genes also exhibited patterns of gene regulation in the [KO + WT N-ras] *vs.* [KO + N-ras-Palm*H*] comparison that were similar to the patterns of gene regulation that had been seen in the microarray, in the empty vector validation experiments, and the rescue experiments that utilized the WT N- and H-ras expression constructs (data not shown).

## Discussion

One of the findings of the current study is that N-ras and H-ras regulated different sets of transcripts in immune cells. Previous results from our laboratory [14], [22] showed that there were differences in the activation of ERK, AKT and other downstream signaling components in immune cells in the presence and absence of N-ras; specifically: the levels and kinetics of AKT, ERK and JNK activation were shown by Western blotting to differ between WT and N-ras KO thymocytes [14]. A more recent study by our group also demonstrated that there were differences in the levels of ERK activation between low-level TCR stimulated Jurkat T-cells that overexpressed WT H-ras and that overexpressed WT N-ras [22]. The differential activation of downstream signaling components, such as: ERK, AKT and JNK, by N-ras and H-ras could partly explain the different gene regulation patterns of the two Ras isoforms in immune cells.

There are two of previous results from our group that are worth discussing in light of the results of the current study. The first is the observation that endogenous H-ras is expressed at much lower levels than endogenous N-ras in Jurkat T-cells and in other T-cell lines [22]. Earlier studies from our group have also demonstrated that H-ras mRNA is expressed at lower levels in both the spleen and thymus than mRNA for N-ras [1]. We do not believe that the differential expression level of H-ras and N-ras can account for the differential pattern of gene regulation that we observed for the two Ras isoforms in immune cells. In previous studies, when H-ras was overexpressed in Jurkat T-cells, activation of H-ras at the Golgi in response to a low-level TCR stimulus was not observed [22]. This result implies that other factors, such as differential membrane compartmentalization, might be responsible for the preferential Golgi activation of N-ras that was seen previously, and might also explain the differential pattern of gene regulation that we observed for the Ras isoforms in the current study.

The second observation that is worthy of comment is that a small but significant difference was previously seen in the size of particular immune cell populations in the thymus and in the spleen of N-ras KO mice [14]. Although differences in the size of some immune cell populations were found in the KO mouse, the size of the CD4^+^CD8^−^ splenocyte population was the same in KO and WT mice [14]. Since CD4^+^ T-cells were used for the second set of microarray experiments in this study, we would not expect to have differences between WT and KO mice that would have impacted our results.

In an earlier study, Santos' group [24] had examined the gene expression profiles in N-ras KO and H-ras KO mouse embryonic fibroblasts (MEFs); a comparison of our results to theirs revealed a number of key differences. First, we found that many more genes were differentially regulated by N-ras and by H-ras than was seen by Santo's group; this might reflect a larger role for the Ras proteins in immune cells as opposed to fibroblasts. Rather than the large differential between the numbers of genes differentially regulated by H-ras and N-ras that Santos' group observed, we found that similar numbers of genes were differentially regulated by N-ras and by H-ras in thymocytes and stimulated CD4^+^ T-cells. In our experiments, there was also an even split between the numbers of genes that were upregulated and that were downregulated by N-ras in both types of immune cells, which is in contrast to Santo's results, where most of the genes differentially regulated by N-ras were downregulated. It is possible that the differences in N-ras- and H-ras-specific transcriptomes between the two studies could be attributed to Ras isoforms playing different roles in immune cells than they do in MEFs, but we can also not rule out that the differences between the results of the two studies could be due to differences in the way the individual microarray experiments were conducted or were analyzed.

We also compared the genes that were differentially regulated by N-ras and H-ras in Santo's study [24] to the genes that we found to be differentially regulated by N-ras and by H-ras in thymocytes. There was little overlap between the differentially regulated genes between the two studies; the handful of genes that were differentially regulated by N-ras and H-ras in both MEFs and in immune cells belonged to ‘general’ cellular pathways such as: signal transduction, cellular transport, or cell cycle regulation.

It is important to note that in the Ras isoform transduction validation experiments, the over-expression of singly palmitoylated, WT N-ras in a N-ras KO T-cell background was able to ‘rescue’ the expression pattern of the four candidate genes, whereas the over-expression of doubly palmitoylated WT H-ras or the doubly palmitoylated N-ras-Palm*H* mutant was not able to recue the expression pattern of downstream genes in these cells. This result extends the observations from a previous study by Perez de Castro, et al. [22], in which it was shown that the palmitoylation state of the Ras isoform determined both their ability to be activated at the Golgi by a low-level TCR stimulus as well as determining the ability of the Ras isoform to elicit downstream signaling in response to a low-level TCR stimulus. In addition, the results of the rescue experiment also integrate well with previous results from our laboratory (Lynch SJ, *et al*., manuscript in preparation) in which it was shown that the palmitoylation state of the Ras isoform determines both their Golgi membrane localization pattern, as well as the ability of the Ras isoform to regulate the expression of specific genes in T-cells in the context of a low-level TCR stimulus.

### Potential Immune Cell Specific Functions of the Four Genes Downstream of N-ras in Stimulated CD4^+^ T-cells

In our second set of microarray experiments, T-cells were consistently treated overnight with a low-level TCR stimulus (1 μg/ml α-CD3e and α-CD28 antibodies) prior to RNA isolation. In order to extrapolate on potential functional roles of the four genes that we found to be differentially regulated by N-ras in this cellular context, it is important to keep in mind the physiological relevance of a low-level TCR stimulus. It has been shown that developing T-cells encounter stimuli that elicit a low-level TCR response during two cellular processes: during positive selection in the thymus, where cells that respond to the presentation of self MHC as if it were a low-level TCR stimulus are selected for survival [25]; and during T-cell affinity maturation in the periphery, where T-cells that respond to antigen stimulation as if it were a low level stimulus (low affinity T cell clones) are actively eliminated through apoptosis [26].

The product of the *Slc9a6* gene is NHE6, a Na^+^/H^+^ ion exchanger that localizes to sorting/recycling endosomes, where it functions in apical surface protein recycling and sorting through a fine-tuning of the luminal pH of the endosomal compartment [27], [28]. In clinical populations, point mutations of *Slc9a6* that target NHE6 for increased degradation are associated with aberrant glutamate/glutamine recycling in polarized neurons, which is believed to contribute to Angelman syndrome-like X-linked mental retardation [29], [30]. The function of NHE6 in lymphocytes is presumably similar to its function in other cell types, although the identity of the proteins whose membrane recycling might be altered by N-ras-induced upregulation of NHE6 expression are not known. It is tempting to speculate that N-ras-mediated induction of *Slc9a6* could be a mechanism for N-ras to regulate either its own PM or Golgi membrane expression or to regulate the localization of membrane proteins associated with T-cell positive selection, such as: components of the TCR receptor or the co-stimulatory receptor complexes.

The *Chst1* gene encodes for the enzyme CHST1 (carbohydrate sulfotransferase 1), a Golgi-localized, transmembrane domain sulfotransferase that sulfonates specific O-linked carbohydrate side chains on lipids and proteins [31], [32]. One well-studied class of proteins modified by CHST1 are the ligands for the L-selectin receptor. The expression of L-selectin receptors are induced on vascular endothelium and on arrested immune cells at sites of tissue inflammation, and cells expressing L-selectin receptors have been shown to serve as docking sites for circulating leukocytes that express cognate L-selectin ligands [33]–[35]. It has been proposed that immune cells expressing L-selectin ligands can induce the subsequent arrest of further circulating immune cells that express L-selectin receptors, thereby amplifying the number of effector cells accumulating at sites of inflammation [34]. We hypothesize that the upregulation of *Chst1* by N-ras might be important for upregulating the expression of functional L-selectin ligands. In this fashion, T-cells that exhibit a low reactivity for self MHCs during T-cell positive selection might not only receive survival signals, but at the same time, might become primed for the effector functions that they will ultimately perform in the periphery.

The *Lars2* gene encodes the LARS2 enzyme (human leucyl-tRNA synthetase 2, mitochondrial), which catalyzes the charging of mitochondrial tRNA^Leu(UUR)^ with leucine during the synthesis of mitochondrial-specific proteins. Altered expression of *Lars2* has been linked to a number of disease states in human populations [36], [37]. Affinity maturation-induced apoptosis of T-cells involves the induction of mitochondrial-specific proteins from the mitochondrial respiratory chain (MRC)-dependent apoptotic pathway, such as: cytochrome *c* oxidase subunit II (COXII) [38], [39]. It is possible that the low-level TCR stimulation paradigm that we used mimics the T-cell signaling that occurs during affinity maturation-induced apoptosis, and an increased induction and expression of the LARS2 enzyme might therefore be responsible for an increased tRNA processing of mitochondrial-specific pro-apoptotic factors, such as COXII.

The *Dntt* gene encodes for the Terminal deoxynucleotidyl transferase (Tdt) enzyme, which catalyzes the addition of non-template nucleotides during V(D)J recombination of the B-cell receptor (BCR) and the TCR during B-cell and T-cell maturation [40]. During the transition from double positive (DP: CD4^+^, CD8^+^) to single positive (SP: CD4^+^CD8^−^ or CD4^−^CD8^+^) thymocytes, *Dntt* is one of several genes, including: *Rag1, Rag2,* and *CD4,* whose expression is epigenetically silenced [41]–[43]. Given the early inactivation of *Dntt*, it was not clear to us how N-ras could be downregulating the expression of *Dntt* in mature, T-cell splenocytes following a low-level TCR stimulation. We initially entertained the possiblity that N-ras might be important for some aspect of the maintenance of silencing of *Dntt* in these cells, however, we later realized that our preliminary analysis of the microarray data did not rule out the possibility that N-ras could be playing a role in the downregulation of *Dntt* at earlier time points, perhaps even during the DP to SP thymocyte transition. A reexamination of the raw microarray data revealed that *Dntt* was not only downregulated by N-ras in the comparison between the [WT + stimulation] and [N-ras KO + stimulation] conditions, but was also similarly downregulated by N-ras in the comparison between the [WT without stimulation] and [N-ras KO without stimulation] conditions. The fact that *Dntt* was downregulated by N-ras regardless of the TCR stimulation state of the cell, left open the possibility that N-ras is important for downregulating *Dntt* expression at an early time point. Our results are therefore consistent with N-ras playing either direct or indirect roles in the establishment *Dntt* locus silencing, although we can not rule out that N-ras regulates later aspects of the heritable maintenance of *Dntt* silencing, i.e. through the regulation of CpG methylation of the *Dntt* locus.

## Supporting Information

Protocol S1 **Protocol Used for the Retroviral Transduction of CD4^+^ T-cells.**
(DOCX)

Table S1 **Forward and reverse primer sets used for each of the 31 candidate genes tested in qRTPCR-based validation experiments.**
(TIF)

Table S2 **Properties of the thirty-one candidate genes that were further tested in qRT-PCR-based validation experiments.** For each of the thirty-one genes, the fold change (F.C.), *p*-value, gene name, gene title, functional annotation category/proposed function, average absolute expression values in the raw microarray data, and median absolute expression values in the raw array data are listed. Note that the eight candidate genes that exhibited similar expression patterns in the WT *vs*. KO microarray comparisons and the qRT-PCR-based, empty vector control validation experiments are indicated in bold.
(XLSX)

Table S3 **(A) 303 genes that were differentially regulated by N-ras in unstimulated thymocytes in a comparison between [WT] vs. [N-ras KO] microarray data sets.** For each gene, the gene symbol, gene title, direction of regulation, fold change (F.C.) of regulation, Genbank accession #, and Affymetrix Probe ID are listed. Note that bold-faced font indicate genes that were also differentially regulated by N-ras in the same cellular context. (B) List of transcripts that were differentially regulated by N-ras in unstimulated thymocytes in a comparison between [WT] vs. [N-ras KO] microarray data sets that represent expressed sequence tags and cDNA and mRNA clones. Overall format of this table is the same as in (A).
(XLSX)

Table S4 **(A) 367 genes that were differentially regulated by H-ras in unstimulated thymocytes in a comparison between [WT] vs. [H-ras KO] microarray data sets.** For each gene, the gene symbol, gene title, direction of regulation, fold change (F.C.) of regulation, Genbank accession #, and Affymetrix Probe ID are listed. Note that bold-faced font indicate genes that were also differentially regulated by N-ras in the same cellular context. (B) List of transcripts that were differentially regulated by H-ras in unstimulated thymocytes in a comparison between [WT] vs. [H-ras KO] microarray data sets that represent expressed sequence tags and cDNA and mRNA clones. Overall format of this table is the same as in (A)
(XLSX)

Table S5 **1652 transcripts that were differentially regulated by N-ras (F.C. ≥1.5, p-value ≤0.05) in CD4^+^ T-cells in a comparison between [WT + stim.] vs. [N-ras KO + stim.] mRNA expression profiling data sets.** For each transcript, the gene symbol, gene title, fold change (F.C.) of regulation, direction of regulation, *p*-value, Entrez Gene #, median absolute expression values in the raw microarray data, average absolute expression values in the raw array data, and the Affymetrix Probe ID are listed.
(TIF)
