# Correction: In TCR-Stimulated T-cells, N-ras Regulates Specific Genes and Signal Transduction Pathways

**DOI:** 10.1371/annotation/f0d2b6b0-7d87-492e-8505-5159c4b5bdd5

**Published:** 2013-09-10

**Authors:** Stephen J. Lynch, Jiri Zavadil, Angel Pellicer

The titles and legends for Supporting Tables S2, S3, S4 and S5 were incorrectly switched. The title and legend currently appearing with Supporting Table S2 belong with Supporting Table S5, the title and legend currently appearing with Supporting Table S3 belong with Supporting Table S2, the title and legend currently appearing with Supporting Table S4 belong with Supporting Table S3, and the title and legend currently appearing with Supporting Table S5 belong with Supporting Table S4. 

