# Supplementary material for: In TCR-Stimulated T-cells, N-ras Regulates Specific Genes and Signal Transduction Pathways
Source: PLoS One. 2013 Jun 3;8(6):e63193. doi: 10.1371/journal.pone.0063193 (PMC3670928; doi:10.1371/journal.pone.0063193)
Supplement: Protocol S1 — Protocol Used for the Retroviral Transduction of CD4+ T-cells. (DOCX) [file pone.0063193.s001.docx]

**Supplementary Protocol 1**

**Protocol Used for the Retroviral Transduction of CD4^+^ T-cells**

Effectene transfection reagent (Qiagen) was used to transfect 293T cells with either the MIGR1, MIGR1-N-Ras or MIGR1-H-Ras expression vectors and a psi ecotropic packaging vector. At 48 hours post-transfection, the retroviral supernatant was isolated, and was filtered through a 0.45 μm filter (Millipore) before being used to infect CD4^+^ T-cells (see below). CD4^+^ T-cell splenocytes were isolated from WT and N-ras KO mice following the protocol described above. CD4^+^ T-cells were spun down and resuspended in T-cell growth media supplemented with 1 μg/ml of purified hamster anti-mouse CD28 antibody and 0.2 μg/ml of purified hamster anti-mouse CD3e antibody, and were plated onto 24-well plates that had previously been coated with goat IgG fraction to hamster IgG. For these experiments, 1 x 10^7^ cells were plated out per experimental condition, with 5 x 10^5^ cells being added per well of a 24 well plate. The CD4^+^ T-cells were incubated at 37^o^C/5% CO_2_ overnight.

Following an overnight incubation, CD4^+^ T-cells were infected with retroviral supernatant via spin infection at 2,500 rpm for 2 hours. Following the spin infection, the pelleted cells were resuspended in T-cell growth media, and were incubated for 48 hours at 37^o^C/5% CO2. Infected cells were then isolated by sorting for GFP in a MoFlo cell sorter. The GFP^+^ transduced cells were resuspended in T-cell growth media supplemented with 1 μg/ml of anti-mouse CD3e and CD28 antibodies, and were incubated overnight in at 37^o^C. The next day, the infected CD4^+^ T-cells were homogenized using Trizol reagent, and total RNA was isolated from these cells using the protocol described in the Material and Methods.
