# Supplementary figures and images for: In TCR-Stimulated T-cells, N-ras Regulates Specific Genes and Signal Transduction Pathways
Source: PLoS One. 2013 Jun 3;8(6):e63193. doi: 10.1371/journal.pone.0063193 (PMC3670928; doi:10.1371/journal.pone.0063193)

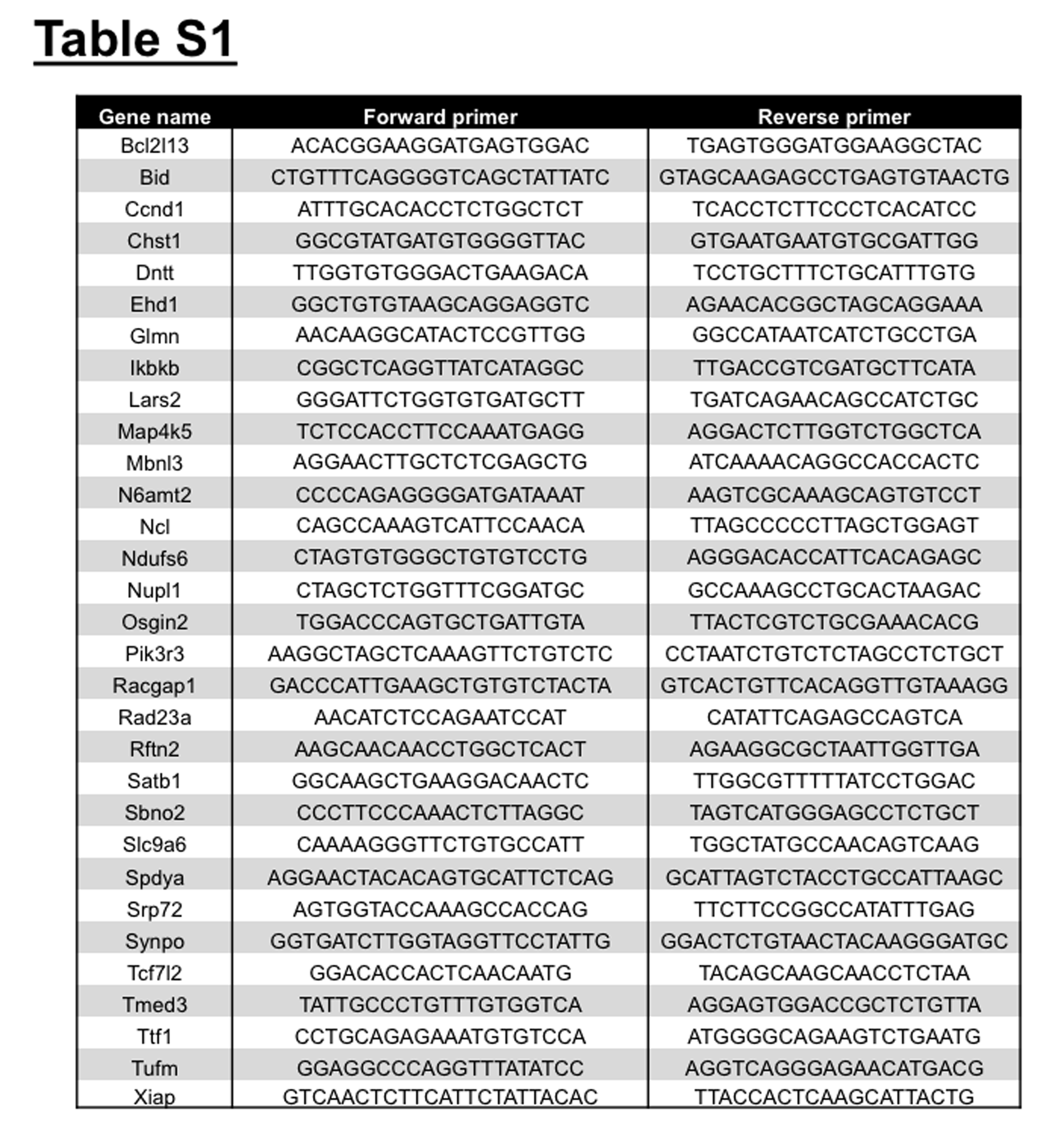

Supplement: Table S1 — Forward and reverse primer sets used for each of the 31 candidate genes tested in qRTPCR-based validation experiments. (TIF) [file pone.0063193.s002.tif]

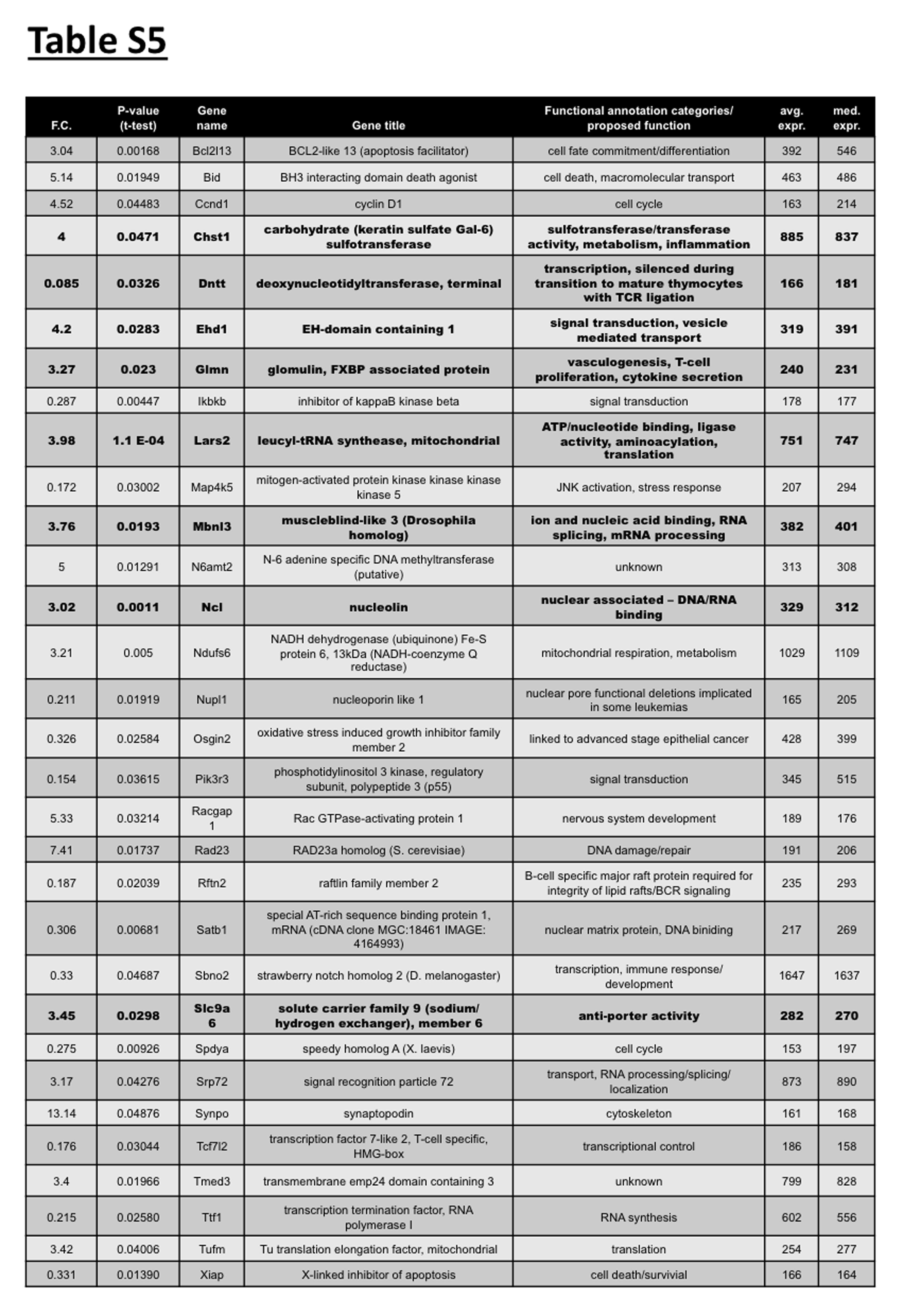

Supplement: Table S5 — 1652 transcripts that were differentially regulated by N-ras (F.C. ≥1.5, p-value ≤0.05) in CD4+ T-cells in a comparison between [WT + stim.] vs. [N-ras KO + stim.] mRNA expression profiling data sets. For each transcript, the gene symbol, gene title, fold change (F.C.) of regulation, direction of regulation, p-value, Entrez Gene #, median absolute expression values in the raw microarray data, average absolute expression values in the raw array data, and the Affymetrix Probe ID are listed. (TIF) [file pone.0063193.s006.tif]
